# The Impact of the Coronavirus Disease 2019 Pandemic on Investor Sentiment—Evidence From A-Share Listed Companies in China

**DOI:** 10.3389/fpsyg.2021.743306

**Published:** 2021-09-13

**Authors:** Yuegang Song, Xiazhen Hao, Zhou Lu

**Affiliations:** ^1^School of Business, Henan Normal University, Xinxiang, China; ^2^School of Economics, Tianjin University of Commerce, Tianjin, China

**Keywords:** investor sentiment, COVID-19 pandemic, difference-in-differences model, financial market, A-share listed companies

## Abstract

In a DID model, this study examines the impact of the coronavirus disease 2019 (COVID-19) pandemic on the investor sentiment in the financial market of China using monthly panel data on newly listed Chinese companies between October 2019 and June 2020. The outbreak of the pandemic is shown to exert a significant negative impact on investor sentiment. A future industry heterogeneity analysis shows that the pandemic has driven up investor sentiment in the pharmaceutical sector while having a significantly negative impact on non-pharmaceutical sectors. The pandemic is shown to have a negative impact on the private sector and foreign-invested sector in China while a significantly positive impact on the state-owned sector. This study contributes to the existing literature on the investigation of how significant the impact of public health emergencies on investor sentiment is.

## Introduction

The 2020 outbreak of the coronavirus disease 2019 (COVID-19) pandemic, a global public health crisis, has hit the Chinese economy and the world economy hard. According to the WHO statistics, the pandemic has swept across more than 200 countries, causing a total of 83 million people worldwide with confirmed infection while killing over 1.8 million people.

The impact of the COVID-19 pandemic on the financial market of China is substantial. Shanghai Composite dipped by around 7.7%, a 5-year single-day low. At the same time, CSI Aggregate Bond rose by about 0.62%, a second decade-high. The forex market and currency market are also hit to various extents. Investor sentiment can reflect the general market trend and market vitality (Yu et al., 2020). Then, does the pandemic exert an impact on investor sentiment? If the answer is yes, then how should we measure the impact? Does investor sentiment vary across firm types and industries? How should China respond to the change? The answers to the above questions may help policymakers take measures to stabilize the financial market and formulate solutions to financial market turmoil. However, there is a minimal existing research literature on the impact of major public health events on investor sentiment. This study is intended to contribute to the understanding of this issue by looking at the impact of the COVID-19 pandemic on investor sentiment in the financial market of China.

This study uses a difference-in-difference (DID) model to study the impact of the COVID-19 pandemic on investor sentiment in the financial market of China. The improved turnover rate is used to measure the investor sentiment on individual stocks. This study focuses on the following variables: trading volume, free float equity, market value, financial indicators, etc., trying to study the impact of major economic shocks on investor sentiment in theA-share market of China. This study has important policy value for economic policies in responding to major public health incidents, stabilizing the A-share market, and safeguarding economic development in China.

The rest of the study is roughly organized as follows. The “Literature review” section reviews the previous studies. The “Model, variable selection, and data sources” section explains the model, variable, and data. The “Empirical analysis and findings” section provides the details of the empirical analyses. The “Conclusion” section concludes.

## Literature Review

### Investor Sentiment Literature

Behavioral finance theories imply a considerable impact of the psychological factors of the investor on the financial market. Existing approaches to measuring investor sentiment include direct proxy, indirect proxy, and text mining. In direct proxy, Brown and Cliff (2005) used a questionnaire survey to understand the outlooks of the investor on market trends. Kenneth and Meir (2000) categorized the investors into three subcategories, namely, large investors, medium-size investors, and small investors. Jiang et al. (2021) regarded the Baidu index as an indicator of investor sentiment and believed that investor sentiment is usually affected by the information provided by the Baidu search engine, which may cause stock prices to fluctuate. As single-indicator-based measurement may be subjected to bias, some scholars constructed an index system to measure investor sentiment. Baker and Wurgler (2006) and Yi and Mao (2009) cited six independent proxy indices to construct the investor sentiment composite index based on principal component analysis.

Individual investor sentiment is measured primarily based on big data analysis methods as data analysis and text mining. Baker and Stein (2004) pointed out that liquidity measured by turnover rate can be an indicator of investor sentiment. Das and Chen (2008) proposed a method of gauging the sentiment of small investors from a web-based message board by comparing the optimistic and pessimistic views. Balke et al. (2017) and Zhang (2019) provided methods to measure sentiment based on surveys and government policies. Yang et al. (2016) used big data mining to measure the investor sentiment index using big data mining from around 900,000 posts issued by listed companies on the website of Eastmoney Securities. Fang et al. (2020) used the data from Baidu, the leading search engine in China, to construct an indicator of investor sentiment for forecasting of returns of the Chinese stock market.

The existing literature also investigates how investor sentiment exerts an impact on the stock market and what factors drive investor sentiment (Delong et al., 1990; Fisher and Statman, 2000). In studying the correlation of asset pricing with investor sentiment, Brown and Cliff (2005) suggested the latter indicator has an impact on the former, i.e., investor sentiment change leads to stock fluctuation. Based on the statistics of the Chinese stock market, Xie and Tang (2021) concluded that positive investor sentiment can drive up the yield rate to some degree and this impact can last for around a year. Gozgor et al. (2019) pointed out that economic policy uncertainty affects gold returns, which in turn affects investor behavior and investor sentiment. During periods of high economic policy uncertainty, especially during the early 2020s and the COVID-19 pandemic, economic policy uncertainty exerts a considerable impact on the financial stock market and affects investment returns (Wu et al., 2021). Li et al. (2017) and Zhang et al. (2021) constructed investment sentiment indicators and analyzed the impact of external shocks on the sentiment of Chinese investors.

### Literature on the Impacts of Major Public Health Events

Since the twenty-first century, there have been five grave pandemics of infectious diseases defined by WHO as major public health events on a global scale or higher. Many scholars have assessed the impact of these events on the growth of the economy as a whole (Brainerd and Sieglar, 2003; Wu, 2003; Hanna and Huang, 2006). Barro et al. (2020), based on the death toll during the 1918–1929 influenza pandemic and the death toll in World War I, estimated the death toll of the COVID-19 pandemic and its economic impact, finding the severely impacted countries registered a 6% drop and an 8% drop in GDP and consumption, respectively. The outbreak of the COVID-19 pandemic interrupts the product supply chain, and the global economy is in face of a recession (Shang et al., 2021). Brem et al. (2021) discussed 10 technologies that play a major role in the COVID-19 crisis and found that technological innovation has a key role in response to the epidemic and subsequent economic recovery. Cai et al. (2021) analyzed the impact of the explosive pandemic on the labor market of China. The results showed that under the impact of the pandemic, overall employment assumed a “V”-shaped trend. Based on the monthly panel data of Chinese provinces and cities, Zhang and Zhu (2021) used DID model to analyze the impact of the pandemic on various industries and concluded that the pandemic has a significant negative impact on international trade and the road freight industry.

External shocks tend to exert a significant impact on the financial stock market (Baker et al., 2020; Zhao et al., 2021). Chen (2020) used the event analysis method to analyze the impact of the pandemic on the stock market of China, and the results showed that the return rate of the market at large had dropped significantly during the pandemic and impacted on different types of firms to varying degrees. Wang et al. (2020) used the panel VAR model and dynamic econometric model to evaluate the impact of the COVID-19 on stock price fluctuation and concluded that fear and anxiety from the pandemic may drive investors to sell stocks and to cause price fluctuation. Sun et al. (2021) studied the impact of investor sentiment during the pandemic on pharmaceutical stock trading in mainland China, Hong Kong, South Korea, Japan, and the United States, and the results showed that ERAs had a significant positive effect on pharmaceutical investment portfolios in these markets.

The related investigations focus on investor sentiment mainly from the perspective of investor attention metrics and the impact of investor sentiment on the financial stock markets; major public health events are studied based on the impact on the economy and the financial market. Few studies employ investor sentiment as an explained variable to study the impact of the pandemic as an external shock on investor sentiment. Our study is intended to make three contributions to the existing literature. First, we introduced the listed firms on the A-share stock market of China as the research object and analyzed the impact of the pandemic on investor sentiment at the firm level. Second, the authors introduced new research methods by considering the pandemic as a quasi-natural experiment and building a DID model intended to assess the impact of the pandemic on investor sentiment. Meanwhile, the propensity score matching-difference-in-difference (PSM-DID) method and the placebo-controlled test were used to conduct a series of robustness tests. Third, the heterogeneity analysis was applied to the model being built to illustrate the impact of the pandemic on investor sentiment based on the firm type and industry differences.

## Model, Variable Selection, and Data Sources

### Model Building

In January 2020, the pandemic first broke out in Wuhan, China and then quickly spread to other cities. However, the Chinese government decisively adopted strict lockdown measures, which resulted in effective control of the spread of the virus. To analyze the impact of the pandemic on domestic investor sentiment, we took the 2020 outbreak as the start time, regarded the pandemic as an external shock, and studied investor sentiment based on a DID model. This model is widely used to evaluate the effects of policies in different pilot regions. Similar to the implementation of policies in different pilot regions, the outbreak of the COVID-19 pandemic in different regions can also be regarded as a quasi-natural experiment.

We considered those Chinese provinces with a cumulative number of confirmed infection cases not <1,000 (Zhang and Zhu, 2021). Listed firms registered after December 2019 in Hubei, listed firms registered after February 2020 in Guangdong, Henan, and Zhejiang, and listed firms registered after March 2020 in Hunan are used as the treatment groups, while listed firms listed in other provinces served as the control groups. The time series of provinces with a cumulative number of confirmed cases over 1,000 are shown in Table 1.

**Table 1:** The time series of confirmed cases with a cumulative number of over 1,000 in the provinces.

**Province**	**Number of confirmed cases in province**	**Number of confirmed cases nationwide**	**Time**
Hubei	7,153	11,791	2020/01
Guangdong	1,349	79,824	2020/02
Henan	1,272	79,824	2020/02
Zhejiang	1,205	79,824	2020/02
Hunan	1,018	81,554	2020/03

The model is as follows:


(1) $$\begin{array}{r} sent_{it}=\alpha_{0}+\beta_{1}post_{it}\times treat_{it}+\gamma X_{it}+\nu_{i}+\mu_{t}+\varepsilon_{it}. \end{array}$$

The explained variable *sent* represents investor sentiment on the individual listed firm of *i* at a specific time (*t*). All the listed firms in the sample fall into four groups. If *post*_*it*_ = 1 and *treat*_*it*_ = 1, the firm falls into the post-pandemic treatment group; if *post*_*it*_ = 0 and *treat*_*it*_ = 1, the firm falls into the pre-pandemic treatment group; if *post*_*it*_ = 1 and *treat*_*it*_ = 0, the firm falls into the post-pandemic control group; and if *post*_*it*_ = 0 and *treat*_*it*_ = 0, the firm falls into the pre-pandemic control group. In this study, the core explanatory variable was *pos*_*t*_*it*_ × *treatit*_, and β_1_ measures the impact of the pandemic on investor sentiment. If β_1_ <0, the pandemic is shown to reduce the turnover rate and make the investors pessimistic; if β_1_>0, the pandemic on its full-scale tends to increase the turnover rate. Besides, *X*_*it*_ is a series of control variables, ν_*i*_ is the individual fixed effects, μ_*t*_ is the time fixed effects, and ε_*it*_ is the random error term.

### Variable Selection

sentiment, used in this study as an explained variable, refers to theoretically inexplicable factors that drive investors make stock market forecasts based on macroeconomics and company data. It can be seen as a specific risk in connection with capital market development and reflects the market forecasts and confidence of investors in their own investment strategy. The turnover rate is an indicator normally used in measuring investor sentiment on an individual stock and is calculated generally as trading volume divided by total equity. However, a vast number of the stocks on the A-share stock market are traded off the exchange. In this study, we improved the approach to measuring investor sentiment on individual stocks. We used turnover divided by free float as the proxy variable for investor sentiment. We do not include (i) the free floats of shareholders who held more than 5% of the outstanding shares of the firm and (ii) the free floats of shareholders who held <5% of the outstanding shares of the firm and whose associated shareholders held 5% of the outstanding shares of the firm. According to the studies of Wang and Wang (2014), Larrain and Urzúa (2013), and Tian et al. (2020), we selected seven control variables, namely, firm size (*size*), independent director percentage (*Indep*), firm age (*lnage*), percentage of holdings of top shareholder (*top1*), market value and book value (*mvba*), enterprise accounting performance (*epa*), and financial leverage (*lev*). Table 2 shows the definitions of the control variables in detail.

**Table 2:** The description and definitions of main variables.

**Variable category**	**Variable name**	**Meaning**	**Calculation method**
Explained variable	Sent	Investor sentiment	Trading volume/free float equity
Explanatory variables	Post	Time of the outbreak	The time when the epidemic occurred, after the new crown pneumonia epidemic event, it was 1, and before it was 0
	Treat	Epidemic shock	For the listed companies in the treatment group, the value of Treat is 1, and the value of other companies is 0
Control variable	Size	Enterprise size	Ln(Enterprise total assets)
	Indep	Proportion of independent directors	Total number of independent directors/total number of board of directors
	Lnage	Business age	Ln(Years of listing)
	Top1	Shareholding ratio of the company's largest shareholder	The largest shareholder's shareholding ratio
	Mvba	Book value	Enterprise market value/total assets
	Epa	Corporate Accounting Performance	Corporate return on equity
	Lev	Corporate financial leverage	Total liabilities/total assets

### Data Source

We used monthly panel data of the firms listed on the A-share stock market of China dated from October 2019 to June 2020 as the initial samples. Since the New Year Holiday and the Chinese New Year fell on January 2020, this study eliminated the possible impact of such factors on the A-share stock market by excluding the data on January 2020. In addition, considering the importance of accuracy and robustness of the research findings, we processed the initial samples by excluding real property developers, financial institutions, ST stocks, ^*^ST stocks, samples with data missing, and samples traded for <15 days per month. Finally, 16,983 observation values were returned. All the statistics are sourced from the GTA CSMAR database.

## Empirical Analysis and Findings

### Descriptive Statistics-Based Analysis

Table 3 shows the descriptive statistics of our selected variables.

**Table 3:** The descriptive analysis.

**Variable**	***N***	**Mean**	**SD**	**Min**	**Max**
Sent	16,983	0.610	0.663	0.005	8.269
Indep	16,983	0.380	0.056	0.250	0.800
Epa	16,983	0.018	0.222	−9.179	1.978
Size	16983	22.260	1.273	18.13	29.710
Lev	16,983	0.410	0.194	0.008	0.978
Top1	16,983	0.316	0.140	0.030	0.882
Mvba	16,983	0.197	0.213	0.004	3.343
Lnage	16,983	2.916	0.296	0	4.111

*Source: CSMAR database*.

### Basic Regression Analysis

First, we made a DID estimation of equation (1). The basic regression results are shown in Table 4. The first column showed the estimates of the time fixed effect and the individual fixed effect. The explanatory variable *post* × *treat* equaled to −0.0552 and was significant at the 1% level, indicating that the outbreak of the pandemic drove down the turnover rates on the A-share stock market and led to pessimistic investor sentiment. The second column, including control variables, shows significant results, which indicate that in those provinces hit harder by the pandemic, the listed firms are subject to a more negative shock on investor sentiment. This was due to waning confidence and an overall pessimistic outlook on the financial market.

**Table 4:** Benchmark model regression results.

**Variables**	**(1)**	**(2)**
	**Sent**	**Sent**
Post × treat	−0.0552^***^	−0.0262^**^
	(0.014)	(0.012)
Indep		0.2613
		(0.164)
Epa		0.0199
		(0.018)
Size		−0.1112^***^
		(0.008)
Lev		0.3592^***^
		(0.054)
Top1		−0.1272^*^
		(0.066)
Mvba		0.9582^***^
		(0.038)
Lnage		−0.2402^***^
		(0.030)
Constant	0.4250^***^	3.2275^***^
	(0.015)	(0.200)
Individual fixed effects	Yes	Yes
Time fixed effects	Yes	Yes
Observations	16,983	16,983
Within_R2	0.1302	0.1686
Between_R2	0.0059	0.1911
Overall_R2	0.0535	0.1552

***, **, and * *denote significance at 1, 5, and 10%, respectively. The SEs are in parentheses*.

## Robustness Checks

### Parallel Trend Test

**Figure 1:**
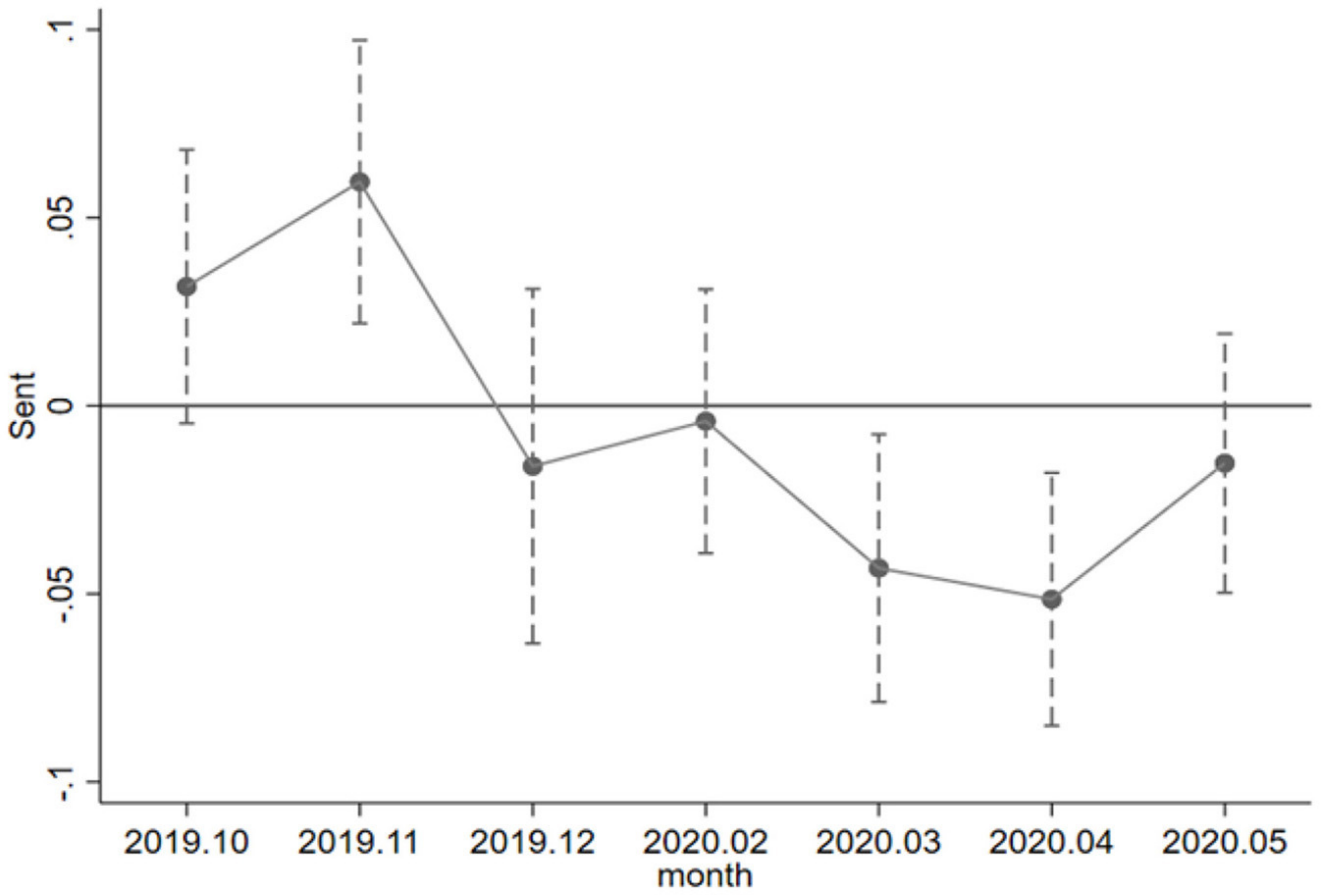
Parallel trend test.

An important assumption for the DID estimation is that the treatment groups and the control groups are assumed to have the same trend without an obvious difference. This study shows that the parallel trend test results are in a 95% CI. As shown in Figure 1, the treatment groups and the control groups had no obvious difference before the pandemic broke out. In early February 2020 when the pandemic broke out, the panic on the A-share stock market of China caused the investors to sell off the holdings in large quantities, and therefore, there was a decline in investor sentiment. After the outbreak, there was a significantly negative, aggravating impact on investor sentiment, which is an important cause for the obvious difference in investor sentiment between the treatment groups and the control groups. In April 2020, the impact on investor sentiment rebounded to some degree thanks to government policy support and effective control measures.

### Heterogeneity Analysis

The impact of the pandemic on investor sentiment may vary with the characteristics of the listed firms; therefore, the impact of the pandemic is analyzed from the perspective of the sector and firm ownership.

The impact of the pandemic on investor sentiment also varies across industries, i.e., the pharmaceutical or non-pharmaceutical sector. This study divided the listed firms on the A-share stock market into the pharmaceutical sector and the non-pharmaceutical sector. As shown in Table 5, when it comes to the pharmaceutical sector, the outbreak of the pandemic had a significantly positive impact on investor sentiment, but when it comes to the non-pharmaceutical sector, there was a significantly negative impact. This result makes sense as the pandemic leads to an upsurge in demand for medical supplies. The government invested more in the pharmaceutical sector, and the investors also expect the sector to be promising.

**Table 5:** Regression results (the pharmaceutical sector vs. the non-pharmaceutical sector).

**Variables**	**(1)**	**(2)**
	**Pharmaceutical industry**	**Non-pharmaceutical industry**
Post × treat	0.1155^***^	−0.0351^***^
	(0.043)	(0.013)
Indep	−0.3468	0.3007^*^
	(0.543)	(0.170)
Epa	0.0521	0.0166
	(0.059)	(0.018)
Size	−0.1198^***^	−0.1057^***^
	(0.029)	(0.009)
Lev	0.3681^**^	0.3545^***^
	(0.174)	(0.056)
Top1	−0.0379	−0.1416^**^
	(0.237)	(0.068)
Mvba	0.2062^**^	1.1044^***^
	(0.087)	(0.042)
Lnage	−0.0479	−0.2308^***^
	(0.107)	(0.032)
Constant	3.0777^***^	3.0568^***^
	(0.726)	(0.208)
Individual fixed effects	Yes	Yes
Time fixed effects	Yes	Yes
Observations	1,533	15,450
Within_R2	0.2099	0.1700
Between_R2	0.0885	0.2116
Overall_R2	0.1440	0.1702

***, **, and * *denote significance at 1, 5, and 10%, respectively. The SEs are in parentheses*.

This study classified the firms traded on the A-share stock market based on firm ownership. Firms involving a state-owned stake were categorized as a state-owned firm, firms involving a privately owned stake were categorized as a privately owned firm, and firms involving a foreign-owned stake were categorized as a foreign-owned firm. In Table 6, columns 1–3 show the regression results of the firms of different ownerships. For the privately owned and foreign-owned firms, investor sentiment has a negative coefficient. In contrast, for the state-owned firms, the investor sentiment coefficient was significantly positive at a 1% level. For the foreign-owned firm, the negative impact of the pandemic on investor sentiment makes sense mainly because the pandemic is an international public health event. The pandemic began to sweep across China in early 2020 and from March 2020, transmitted on a large scale to other countries. The pandemic worsened quickly and countries worldwide took emergency measures in response. Hence, the world economy faced a recession, international demand shrank by a large margin, and import and export markets of China suffered heavily. Therefore, the investors had a generally prudential, or pessimistic, attitude to invest in these firms. Privately owned firms were generally weak in risk resistance, so they were the most impacted. In quite a few provinces, the SMEs went broke under heavy impact. Compared with the privately owned firm and the foreign-owned firm, the state-owned firm was less hit by the pandemic because of government backing as an advantage. When the panic about the pandemic enshrouded the whole market, the market investors chose to buy in more stocks of the state-owned firm, hence its high turnover rate. The investors were, in themselves, optimistic about the state-owned firm. Therefore, the ability of the privately owned firm and the foreign-owned firm to adapt to external shocks should be improved. When a major crisis breaks out, the government should increase its support for such enterprises. The enterprises themselves should increase investment in Science and Technology, promote technological innovation, improve their own anti-risk capabilities, and increase investor confidence in them.

**Table 6:** Ownership regression results (state-owned firm vs. privately owned firm vs. foreign-owned firm).

**Variables**	**(1)**	**(2)**	**(3)**
	**State-owned firm**	**Privately owned firms**	**Foreign-owned firm**
Post × treat	0.1137^***^	−0.0431^***^	−0.0370
	(0.015)	(0.016)	(0.046)
Indep	0.1316	0.2105	0.0294
	(0.162)	(0.241)	(0.623)
Epa	−0.0640	0.0188	0.0081
	(0.054)	(0.025)	(0.034)
Size	−0.0543^***^	−0.1287^***^	−0.0861^***^
	(0.008)	(0.014)	(0.032)
Lev	0.3196^***^	0.4374^***^	0.1071
	(0.061)	(0.075)	(0.193)
Top1	−0.2607^***^	−0.0322	−0.0540
	(0.070)	(0.098)	(0.214)
Mvba	1.1059^***^	1.0299^***^	0.5097^***^
	(0.081)	(0.050)	(0.097)
Lnage	−0.0270	−0.1973^***^	−0.6222^***^
	(0.035)	(0.043)	(0.110)
Constant	1.4236^***^	3.4446^***^	4.0787^***^
	(0.219)	(0.325)	(0.786)
Individual fixed effects	Yes	Yes	Yes
Time fixed effects	Yes	Yes	Yes
Observations	4,319	11,070	1,594
Within_R2	0.0690	0.1879	0.1069
Between_R2	0.2017	0.1231	0.2001
Overall_R2	0.1413	0.1227	0.1328

***, **, and * *denote significance at 1, 5, and 10%, respectively. The SEs are in parentheses*.

### Placebo-Controlled Test

**Figure 2:**
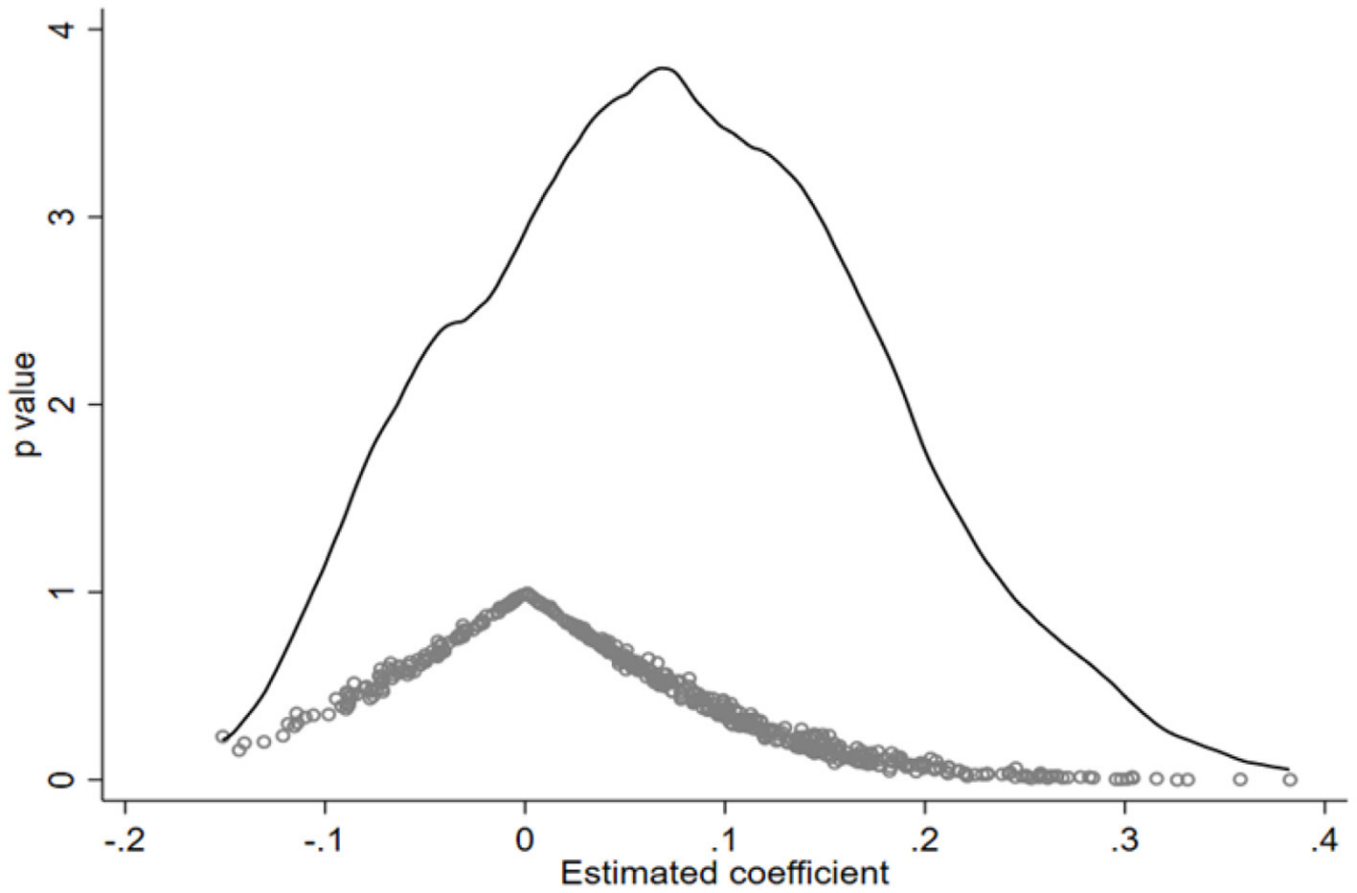
Placebo-controlled test.

To test if investor sentiment is driven by other unobservable factors, a certain number of groups were sampled on a random basis for the purpose of the robustness test. In this study, 500 random samplings were conducted and for each sampling, some of the provinces sampled served as the treatment groups and randomly sampled months between October 2019 and April 2020 served as the time of the outbreak. Our analysis gives the estimated coefficient of investor sentiment as a dependent variable. Based on the basic regression of statistics as shown in Table 4, the estimated coefficient with investor sentiment as the dependent variable was −0.0552, obviously different from the coefficient returned by the placebo-controlled test as shown in Figure 2. Therefore, the existence of other unobservable factors on investor sentiment was insignificant.

### PSM-DID Test

**Figure 3:**
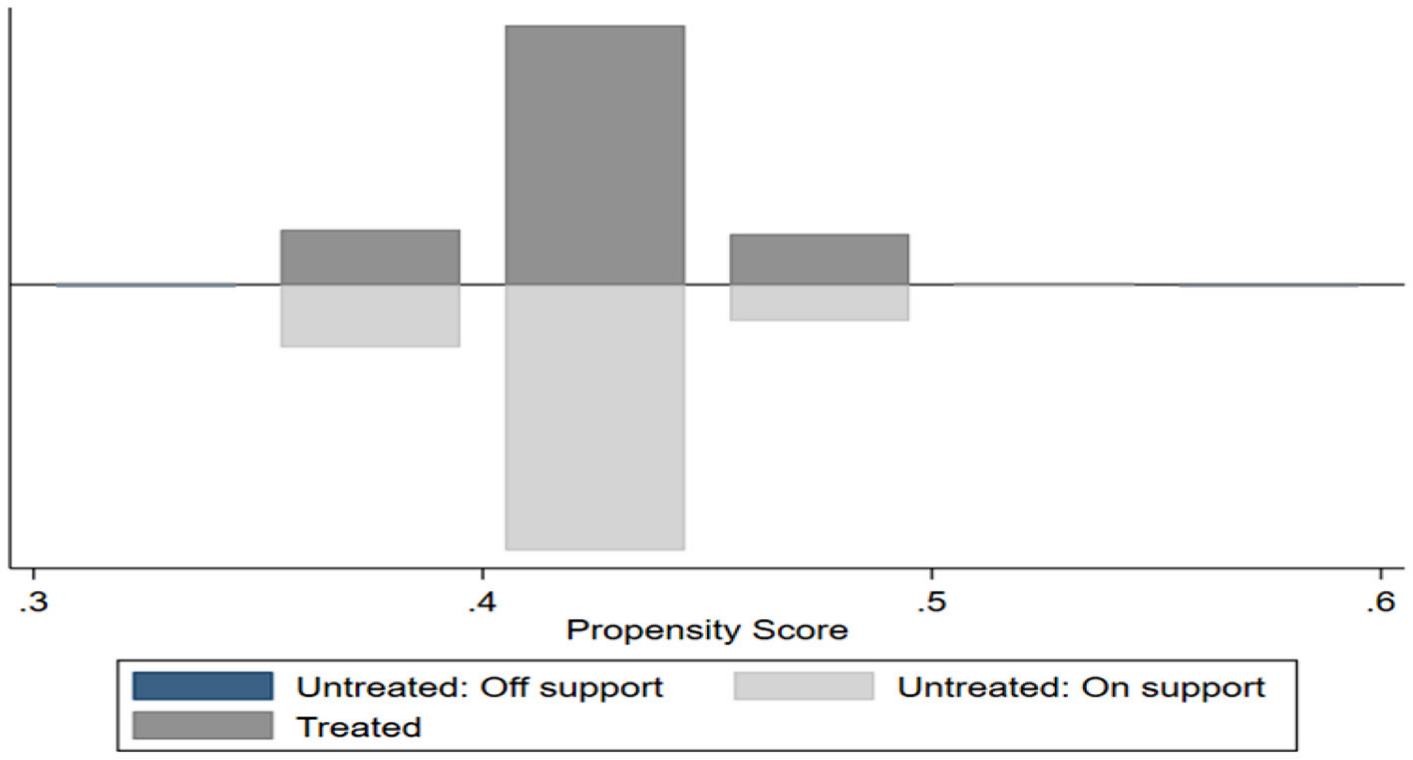
Overlapping assumption test.

This study conducted a PSM analysis of investor sentiment to reduce the selection bias associated with sampling. The various matching variables, i.e., *lnage, size*, the debt-to-asset ratio, and *mvba*, were used as a basis for PSM to select appropriate control groups, and the matched samples underwent DID estimation. An important assumption for PSM is the fulfillment of overlapping one. If both treatment groups and control groups have high propensity scores, it points to a high degree of overlap and the effectiveness of the PSM model, and the reverse is true. Figure 3 shows the results of the overlapping assumption test. There was an overlap of the treatment groups with the control groups, i.e., the overlapping assumption was fulfilled. Also, addressing selection bias with PSM has to fulfill the balance assumption, i.e., except for investor sentiment, there should be no significant difference between the treatment groups and the control groups. It is generally supposed that a post-matching SD <20% points to a good matching effect. Table 7 shows the balancing test results, which shows that the post-matching SDs of the sample variables dropped below 20%, indicative of the fulfillment of the balancing assumption.

**Table 7:** Balancing assumption test.

**Covariate**	**Unmatched U/matched M**	**Treatment group mean**	**Control group mean**	**%bias**	***t* value**	***p* value**
Lnage	U	2.9098	2.9203	−3.5	−2.29	0.022
	M	2.9098	2.9085	0.4	0.26	0.795
Size	U	22.203	22.293	−7.1	−4.59	0.000
	M	22.203	22.177	1.9	1.17	0.241
Lev	U	0.41154	0.40863	1.5	0.97	0.332
	M	0.41154	0.4084	1.6	0.98	0.327
Mvba	U	0.20247	0.1922	4.8	3.10	0.002
	M	0.20247	0.19979	1.3	0.75	0.455

With the above overlapping assumption test and balancing test, a PSM-robustness test was conducted. 1:3 nearest neighbor matching, radius matching, and kernel matching were implemented. Table 8 shows the relative regression results, which shows that the coefficient of the variable post × treat remained significantly at a 1% level.

**Table 8:** Propensity score matching test (nearest neighbor matching vs. radius matching vs. kernel matching).

**Variables**	**(1)**	**(2)**	**(3)**	**(4)**	**(5)**	**(6)**
	**Nearest neighbor matching**	**Nearest neighbor matching**	**Radius matching**	**Radius matching**	**Kernel matching**	**Kernel matching**
Post × treat	−0.0556^***^	−0.0268^**^	−0.0556^***^	−0.0270^**^	−0.0552^***^	−0.0262^**^
	(0.014)	(0.012)	(0.014)	(0.012)	(0.014)	(0.012)
Indep		0.2513		0.2591		0.2613
		(0.164)		(0.164)		(0.164)
Epa		0.0211		0.0566^**^		0.0199
		(0.018)		(0.024)		(0.018)
Size		−0.1123^***^		−0.1118^***^		−0.1112^***^
		(0.008)		(0.008)		(0.008)
Lev		0.3700^***^		0.3666^***^		0.3592^***^
		(0.054)		(0.054)		(0.054)
Top1		−0.1330^**^		−0.1295^**^		−0.1272^*^
		(0.066)		(0.066)		(0.066)
Mvba		0.9709^***^		0.9605^***^		0.9582^***^
		(0.038)		(0.038)		(0.038)
Lnage		−0.2408^***^		−0.2401^***^		−0.2402^***^
		(0.030)		(0.030)		(0.030)
Constant	0.4250^***^	3.2526^***^	0.4251^***^	3.2386^***^	0.4250^***^	3.2275^***^
	(0.015)	(0.202)	(0.015)	(0.200)	(0.015)	(0.200)
Individual fixed effects	Yes	Yes	Yes	Yes	Yes	Yes
Time fixed effects	Yes	Yes	Yes	Yes	Yes	Yes
Observations	16,970	16,970	16,977	16,977	16,983	16,983
Within_R2	0.1302	0.1690	0.1303	0.1690	0.1302	0.1686
Between_R2	0.0057	0.1910	0.0059	0.1913	0.0059	0.1911
Overall_R2	0.0534	0.1556	0.0535	0.1557	0.0535	0.1552

***, **, and * *denote significance at 1, 5, and 10%, respectively. The SEs are in parentheses*.

## Conclusion

To evaluate how and to what extent the COVID-19 pandemic exerts an impact on investor sentiment, this study conducted research on the performance of the A-share stock market of China during the pandemic by doing a quasi-experiment based on the pandemic. Besides, the DID model was used to investigate the impact of the arrived at three conclusions. First, the pandemic drove down the turnover rate on the A-stock market and made the investors pessimistic, so those currently without holdings did not involve themselves in market trading. Second, the pandemic impacted negatively on investor sentiment and brought it down significantly when it comes to the privately owned firms and the foreign-owned firms; in contrast, the state-owned firms attracted many individual investors on the strength of their unique advantages. Third, the outbreak of the pandemic drew broad attention to the pharmaceutical industry from the government and people, so it boosted up investor sentiment in the pharmaceutical industry while having a significantly negative impact on the non-pharmaceutical industry.

In general, considering the pandemic as an external shock that exerts a substantial impact on investor sentiment, the Chinese government and the competent regulatory agencies should implement regulations and establish an early risk warning system. Education and protection should be provided for individual investors to maintain financial security and stability. Based on the aforesaid research findings, this study proposed the following three policy recommendations. First, an early risk warning mechanism and a set of emergency measures should be introduced to the A-share stock market, so in the event of the outbreak of major incidents, i.e., the COVID-19 pandemic, market risk associated with investor sentiment fluctuations can be precluded; besides, appropriate regulatory intervention should be made to limit irrational A-share stock market trading and make the stock market stabler. Second, the competent regulatory agency should educate the investors pertinently, improve their expectations, and guide them to respond reasonably to the external shock of the market. Investor sentiment is an important factor that causes financial stock market volatility under the pandemic. Therefore, psychological monitoring or emotional management intervention during a pandemic outbreak helps people not only to identify mental health problems but also to control their emotions and avoid unreasonable investment behaviors. Third, the impact of the pandemic on investor sentiment should be looked at reasonably. In general, although there was a negative impact on investor sentiment, the pandemic was inversely correlated with the pharmaceutical industry and the state-owned firms, i.e., investigation of investor sentiment at the firm level could be important. Stringent pandemic control measures should be combined with other measures in favor of effective optimistic investor sentiment.

## Data Availability Statement

The original contributions presented in the study are included in the article/supplementary material, further inquiries can be directed to the corresponding author/s.

## Author Contributions

YS: conceptualization, methodology, formal analysis, writing—original draft preparation, and funding acquisition. XH: data processing, formal analysis, and writing—original draft preparation. ZL: methodology, project management, and funding acquisition. All authors contributed to the article and approved the submitted version.

## Funding

This study obtained the financial support of the National Social Science Foundation (the impact of heterogeneity of regional trade in service agreements on the reconstruction of the global value chain of the manufacturing industry in China; Project No: 20BJY091) and the innovation team project of Philosophy and Social Sciences in Colleges and Universities of Henan Province (coordinated development of urban and rural areas and rural revitalization; Project No: 2021-CXTD-04).

## Conflict of Interest

The authors declare that the research was conducted in the absence of any commercial or financial relationships that could be construed as a potential conflict of interest.

## Publisher's Note

All claims expressed in this article are solely those of the authors and do not necessarily represent those of their affiliated organizations, or those of the publisher, the editors and the reviewers. Any product that may be evaluated in this article, or claim that may be made by its manufacturer, is not guaranteed or endorsed by the publisher.
